# Towards a Safer, More Randomized Lentiviral Vector Integration Profile Exploring Artificial LEDGF Chimeras

**DOI:** 10.1371/journal.pone.0164167

**Published:** 2016-10-27

**Authors:** Lenard S. Vranckx, Jonas Demeulemeester, Zeger Debyser, Rik Gijsbers

**Affiliations:** 1 Laboratory for Molecular Virology and Drug discovery, KU Leuven, Belgium; 2 Laboratory for Viral Vector Technology and Gene Therapy, KU Leuven, Belgium; 3 Leuven Viral Vector Core, KU Leuven, Belgium; Mayo Clinic Rochester, UNITED STATES

## Abstract

The capacity to integrate transgenes into the host cell genome makes retroviral vectors an interesting tool for gene therapy. Although stable insertion resulted in successful correction of several monogenic disorders, it also accounts for insertional mutagenesis, a major setback in otherwise successful clinical gene therapy trials due to leukemia development in a subset of treated patients. Despite improvements in vector design, their use is still not risk-free. Lentiviral vector (LV) integration is directed into active transcription units by LEDGF/p75, a host-cell protein co-opted by the viral integrase. We engineered LEDGF/p75-based hybrid tethers in an effort to elicit a more random integration pattern to increase biosafety, and potentially reduce proto-oncogene activation. We therefore truncated LEDGF/p75 by deleting the N-terminal chromatin-reading PWWP-domain, and replaced this domain with alternative pan-chromatin binding peptides. Expression of these LEDGF-hybrids in LEDGF-depleted cells efficiently rescued LV transduction and resulted in LV integrations that distributed more randomly throughout the host-cell genome. In addition, when considering safe harbor criteria, LV integration sites for these LEDGF-hybrids distributed more safely compared to LEDGF/p75-mediated integration in wild-type cells. This approach should be broadly applicable to introduce therapeutic or suicide genes for cell therapy, such as patient-specific iPS cells.

## Introduction

**Fig 1:**
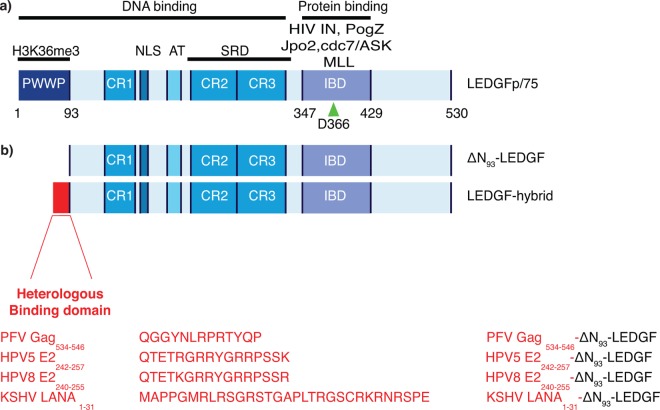
Schematic representation of the LEDGF/p75 domain structure and artificial LEDGF-hybrids. (a) LEDGF/p75 contains a C-terminal protein-binding domain, coined Integrase Binding Domain (IBD) responsible for HIV-IN interaction. Several endogenous proteins like Jpo2, PogZ and MLL bind to the same interface. At its N-terminal end carries multiple chromatin interacting domains, the PWWP domain, the AT hook-like domain (AT) and three charged regions (CR1, 2, 3). D366 is a pivotal amino acid involved in HIV-IN interaction (arrowhead). Mutation to Asn (D366N) abolishes HIV-IN interaction. The lower panel (b) depicts the different LEDGF-hybrids, PFV Gag_534-546_-ΔN_93_-LEDGF, HPV5 E2_242-257_-ΔN_93_-LEDGF, HPV8 E2_240-255_-ΔN_93_-LEDGF and LANA_1-31_-ΔN_93_-LEDGF respectively. Numbers indicate the different amino acid residues. AT, AT-Hook; CR, Charged Region; SRD, Supercoiled Recognition Domein; IBD, Integrase Binding Domain; PWWP, Pro-Trp-Trp-Pro Domain; PFV, Prototype foamy virus; LANA, Latency associated nuclear antigen; HPV, Human papilloma virus; LEDGF, Lens epithelium-derived growth factor; NLS, Nuclear localization signal.

The capacity to integrate transgenes into the host cell genome makes retroviral vectors (RV) an interesting tool for gene therapeutic applications as stable insertion of transgenes into the genome ensures long-term expression. Use of RV-mediated gene transfer resulted in successful cure of several monogenic, primary immunodeficiency disorders [1–3]. Yet, stable insertion occasionally altered endogenous gene regulation resulting in insertional mutagenesis. Due to this major setback 5 out of 19 treated patients developed leukemia in otherwise successful clinical gene therapy trials for X-SCID and 2 out of 2 patients treated for X-CGD acquired myelodysplastic syndrome [3–6]. Both trials employed murine leukemia virus (MLV)-based gammaretroviral vectors (γRV) that integrate in close proximity to gene regulatory regions [7–9] and resulted in transcriptional deregulation due to up-regulated *LMO2* expression [10–13]. Similar reports on insertional mutagenesis were published after integration of γRV near *CCDN2*, *BMI1* and *EVI1* [14,15]. Despite improvements in vector design (e.g. self-inactivating (SIN) vectors) their use is still not risk-free [3,4,6,14–16], which shifted attention from yRV towards HIV-derived lentiviral vectors (LV). Even though LV display a more favorable integration pattern, induction of aberrant splicing [17,18] and insertional mutagenesis remain a major concern, as clonal expansion was observed in a gene therapy trial for β-thalassemia [19]. In addition, two recent independent studies revealed clonal expansion in HIV-1 infected patients on antiretroviral therapy due to HIV-1 virus triggered insertional mutagenesis [20,21]. Retroviral integration is a non-random process which is, depending on the viral genus, associated with specific chromatin marks and genomic features [22–24]. yRV predominantly integrate in the vicinity of gene regulatory regions, whereas LV preferably target the body of active transcription units [10,25]. Integration is catalyzed by the viral integrase (IN), whereas integration site choice bias is attributed to the cellular chromatin readers that are co-opted by the viral IN. Whereas the bromodomain and extra-terminal domain (BET) family of proteins (BRD2, 3 and 4) guide MLV integration [26–28], LV integration is directed by Lens epithelium-derived growth factor p75 (LEDGF/p75) [29,30]. Both function as molecular tethers in the cell, combining a chromatin-binding and a protein-interacting region (reviewed in [31]). For LEDGF/p75 (Fig 1A), the chromatin-binding part contains an N-terminal Pro-Trp-Trp-Pro (PWWP) epigenetic reader domain (aa 1–93), recognizing H3K36me3 chromatin marks [32–36], and a set of DNA-binding motifs (Fig 1A, [37,38]). Together, these elements allow LEDGF/p75 to explore the chromatin in a dynamic scan-and-lock fashion [39]. Even though its cellular role is not fully understood, it is clear that LEDGF/p75 acts as a molecular hub for a variety of endogenous proteins next to the lentiviral integrase (Fig 1A) [40,41]^,^[42]^,^[43]^,^[44]. All these proteins, including the lentiviral integrase, bind the C-terminal Integrase-Binding Domain (IBD, aa 347–429; Fig 1A) of LEDGF/p75. We and others showed that replacement of the N-terminal LEDGF/p75 DNA-binding region (aa 1–325) with alternative DNA-binding domains retargets LV integration towards genomic loci bound by these domains [35,45–47]. Fusion of the heterochromatin binding Chromobox protein homolog 1 (CBX1) to the IN-binding C-terminal end of LEDGF/p75 shifted LV integration into the cognate H3K9me_x_-marked chromatin environment, pericentric heterochromatin and intergenic regions [46]. Despite integration in regions enriched in epigenetic marks associated with gene silencing, transgene expression remained efficient and resulted in successful phenotypic correction in a cell model for X-CGD [48].

**Fig 2:**
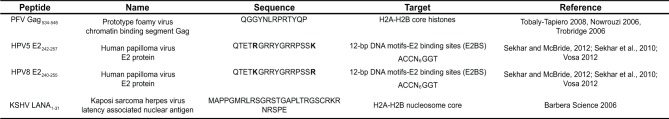
Peptide characteristics. Fig showing the acronyms, aa-sequences and binding characteristics of the peptides used to generate the artificial LEDGF tethers. PFV, Prototype foamy virus; HPV, Human papilloma virus; KSHV, Kaposi’s sarcoma herpes virus; LANA, Latency associated nuclear antigen.

Here we aimed at developing a LEDGF-based tether that results in a more random integration pattern to reduce the overall risk of insertional mutagenesis [11,49–51]. First, we truncated LEDGF/p75 by deleting the N-terminal chromatin-reading PWWP domain that binds H3K36me3 marks directing LEDGF/p75 into the body of active transcription units (Fig 1A and 1B). In addition, we replaced the PWWP-domain with three alternative viral protein domains and motifs, described in literature as pan-chromatin recognition peptides since they bind cellular chromatin without sequence specificity (Fig 1B; Fig 2). Several viruses reside as an episomal DNA genome in host cells, and evolved strategies to persist during mitosis through defined chromatin binding motifs. The spumavirus, Prototype Foamy Virus (PFV), contains a 13-amino acid motif in the group-specific antigen (Gag) binding the H2A/H2B core nucleosome [52–54]. Likewise, the Kaposi Sarcoma-associated Herpes Virus (KSHV) genome is tethered to the nucleosomal core via a chromatin binding sequence (CBS) at the N-terminal end of the latency-associated nuclear antigen protein (LANA) [55]. Finally, in the Beta-Papillomaviruses (PV) a conserved motif in the E2 hinge promotes binding to chromatin and mitotic chromosomes of the invaded cell [56–58]. Following the generation of stable cells lines, we monitored LV integration preferences and evaluated integration sites based on safe harbor region criteria [59] and determined a genotoxicity profile.

## Materials and Methods

### Generation of stable cell lines

SIV-based vector transfer plasmids (pGAE) were a kind gift of D. Nègre (Laboratoire de Vectorologie Rétrovirale et Thérapie Génique, INSERM U412, IFR 74, Ecole Normale Supérieure de Lyon, Lyon, France). A lentiviral vector carrying CMV promoter driving a Zeocin resistance gene and a LEDGF specific miRNA-based shRNA was described earlier [60] and used to generate stable LEDGF_KD_ cells. All LEDGF/p75 hybrid expression constructs were cloned into the pGAE backbone and cloning steps sequence verified.

➢ *Cloning of* Δ*N_93_-LEDGF and* Δ*N_93_-LEDGF_D366N_ controls for the LEDGF* Δ*N_93_-LEDGF fusions*

pGAE_SFFV_ZnF4_ Δ***N***_***93***__BC_I_BsdR_WPRE cl.9 and pGAE_SFFV_ZnF4_ Δ***N***_***93***_ BC_D366N_I_BsdR_WPRE cl.3 were digested using *BglII* & *XhoI*. Ligation of the synthetic adaptor Ad_BglIIKO_AgeI_kozak generated pGAE_SFFV_ Δ***N***_***93***__BC_I_BsdR_WPRE cl. E and pGAE_SFFV_ Δ***N***_***93***__BC_D366N_I_BsdR_WPRE cl. 5. We further refer to the controls as ΔN_93_-LEDGF and ΔN_93_–LEDGF_D366N_ respectively.

➢ *Cloning of LEDGF* Δ*N_93_-LEDGF and* Δ*N_93_-LEDGF_D366N_ hybrids*

pGAE_SFFV_ZnF4_ Δ***N***_***93***__BC_I_BsdR_WPRE cl.9 and pGAE_SFFV_ZnF4_ Δ***N***_***93***_ BC_D366N_I_BsdR_WPRE cl.3 were digested using *BglII* & *XhoI*. Ligation of the synthetic adaptors (for adaptor sequences see S1 Fig) LANA31, PFVCBS13, HPV5E2_16 and HPV8E_216 generated

- pGAE_SFFV_LANA_1-31__ ΔN_93__BC_I_BsdR_WPRE cl.A9
- pGAE_SFFV_LANA_1-31__ ΔN_93__BC_I_BsdR_WPRE cl.H
- pGAE_SFFV_ PFVCBS13_ ΔN_93__BC_I_BsdR_WPRE cl.13
- pGAE_SFFV_ PFVCBS13_ ΔN_93__BC_I_BsdR_WPRE cl.4
- pGAE_SFFV_ HPV5E2_16 _ ΔN_93__BC_I_BsdR_WPRE cl.19
- pGAE_SFFV_ HPV5E2_16 _ ΔN_93__BC_I_BsdR_WPRE cl.8
- pGAE_SFFV_ HPV8E2_16 _ ΔN_93__BC_I_BsdR_WPRE cl.23
- pGAE_SFFV_ HPV8E2_16 _ ΔN_93__BC_I_BsdR_WPRE cl.11

All cloning steps were confirmed by restriction digest and sequencing.

### Cell culture

All cells were grown in a humidified atmosphere containing 5% CO_2_ at 37°C. HeLaP4 310 LEDGF/p75 depleted cells ([46], further referred to as LEDGF_KD_ cells) were grown in Dulbecco’s modified Eagle’s medium (DMEM; GIBCO-BRL, Merelbeke, Belgium) supplemented with 5% v/v heat inactivated fetal calf serum (FCS; Sigma-Aldrich, Bornem, Belgium), 0.005% w/v gentamicin (GIBCO), 0.05% w/v geneticin (GIBCO) and 0.01% w/v zeocin (Life Technologies, Ghent, Belgium). These cells are monoclonal LEDGF_KD_ cells, derived from HeLaP4 cells (gift from P. Charneau, Institut Pasteur, Paris, France). HelaP4 cells were grown on DMEM (GIBCO) supplemented with 5% v/v heat inactivated fetal calf serum (FCS; Sigma-Aldrich), 0.005% w/v gentamicin (GIBCO) & 0.05% w/v geneticin (GIBCO). HEK 293T cells (gift from O. Danos, Evry, France) were cultured in DMEM medium (GIBCO) with 8% v/v heat inactivated FCS (Sigma-Aldrich) and 0.005% w/v gentamicin (GIBCO). SupT1 cells were cultured in Roswell Park Memorial Institutes medium (RPMI, GIBCO-BRL, Merelbeke, Belgium) supplemented with 10% v/v heat inactivated fetal calf serum FCS (Sigma-Aldrich, Bornem,Belgium) and 0.005% w/v gentamicin (GIBCO). Nalm pre-B cells were cultured in RPMI (GIBCO) with 10% v/v heat inactivated FCS (Sigma-Aldrich) and 0.005% w/v gentamicin (GIBCO).

### Retroviral vector production (SIV-based) and transduction

Lentiviral vector production was performed as described earlier [61]. Briefly, for the generation of vesicular stomatitis virus glycoprotein (VSV-G) pseudo-typed SIV-based lentiviral vectors, HEK 293T cells were transfected with the packaging plasmid specific for SIV (pAd_SIV3+; gift from D. Nègre, Lyon, France), the envelope plasmid encoding VSV-G (pLP-VSVG #646 B, from Invitrogen) and respective transfer plasmids, using polyethylenimine (PEI; Polysciences, Amsterdam, The Netherlands). After collecting the supernatant, the medium was filtered using a 0.45 μm filter (Corning Inc., Seneffe, Belgium) and concentrated using a Vivaspin 15 50,000 MW column (Vivascience, Bornem, Belgium). The vector containing concentrate was then aliquoted per 50 μl and stored at -80°C. Stable cell lines expressing a LEDGF hybrid were generated by transduction of polyclonal LEDGF/p75_KD_ cells with SIV-based vectors and subsequent selection with 0,0003% w/v blasticidin (Invitrogen). For lentiviral transduction experiments (LV eGFP T2A fLuc) cells were transduced ON. 72 hours post-transduction cells were harvested when 90% confluent and used for eGFP FACS-analysis or luciferase activity. The remainder of the transduced cells was further cultivated for at least 20 days to eliminate non-integrated DNA and submitted for integration site sequencing.

### Immunocytochemistry and Laser scanning microscopy

Cells were transfected using Lipofectamine 2000 (Life Technologies, Merelbeke, Belgium) as described earlier [62]. LEDGF-hybrids were detected with the primary polyclonal rabbit anti-LEDGF_480-530_ antibody (A300-848a; 1/500; Bethyl Laboratories-Imtec Diagnostics N.V., Antwerpen, Belgium) and secondary polyclonal goat anti-rabbit antibody (1/500 in PBS, goat-αRb488; Bethyl Laboratories-Imtec Diagnostics N.V., Antwerpen, Belgium). Confocal images were acquired using an LSM 510 META imaging unit (Carl Zeiss, Zaventem, Belgium). Alexa-488 was excited at 488 nm (AI laser), mRFP at 543 (HeNe laser) and DAPI at 790 nm (Spectra-physics Mai Tai laser; Spectra Physics, Mountain View CA). After the main beam splitter (HFT KP 700/543 for mRFP, HFT UV/488/543/633 for eGFP, and HFT KP650 for DAPI) a secondary dichroic beam splitter was used to divide the fluorescence signal (NFT 490 for eGFP, NFT 545 for mRFP). Distinct signals were directed to different detectors and data analysis was performed with the LSM image browser. Overlay images were obtained using ImageJ freeware.

### Western Blot

Protein concentration of 1% SDS (AppliChem, Leuven, Belgium) protein extracts sheared with a 27 G needle (Terumo, Leuven, Belgium) was determined using a bicinchoninic acid (BCA) protein assay (Pierce, Aalst, Belgium). Proteins were separated on a 12.5% w/v SDS-polyacrylamide gel and transferred to a polyvinylidene difluoride membrane (PVDF; BioRad) using an XCell SureLock electrophoresis system (Invitrogen). LEDGF-hybrids were detected using 1/2.000 polyclonal rabbit anti-LEDGF_480–530_ antibody (A300-848a; Bethyl Laboratories-Imtec Diagnostics N.V., Antwerpen, Belgium) and 1/5 000 secondary antibody (polyclonal goat anti-rabbit antibody coupled with horse radish peroxidase (HRP); Dako). Chemiluminescence was measured using a ECL plus western blotting detection kit (Amersham Biosciences, Roosendaal, The Netherlands). Equal loading was verified with a primary monoclonal antibody directed to α-tubulin (mouse, 1/10 000, 1 h at room temperature; T5168, Sigma-Aldrich) and secondary antibody in blocking buffer (1/10 000, polyclonal goat-anti mouse labelled with HRP; Dako). Visualization was done by chemiluminescence (Pierce ECL Western Blotting Substrate, Thermo scientific).

### Luciferase assay

Cells were transduced with LV eGFP T2A fLuc and lysed with 70 μl of lysis buffer (50 mmol/l Tris pH 7.5, 200 mmol/l NaCl, 0.2% NP40, 10% glycerol). FLuc activity was determined using the ONE-glo luciferase assay system according to the manufacturers protocol (Promega, Leiden, The Netherlands) and normalized to the total protein concentration in order to correct for differences in metabolic state. The total protein concentration was measured in parallel using a bicinchoninic acid (BCA) protein assay (Pierce, Aalst, Belgium).

### Flow cytometric analysis

Cells were transduced with LV eGFP T2A fLuc and harvested when 95% confluent. eGFP/YFP fluorescence was monitored by Flow cytometric analysis (FACS, Fluorescence activated cell sorting) using a FACSCalibur flow cytometer (BD Biosciences, Erembodegem, Belgium). Data analysis was performed with the CellQuest Pro software (BD Biosciences, Erembodegem-Aalst, Belgium). The percentage of eGFP positive cells (% of gated cells) multiplied by the mean fluorescence intensity (MFI) is further referred to as overall transduction efficiency.

### Integration site amplification and sequencing

Transduced HeLaP4 cells were further cultivated for at least 20 days to eliminate non-integrated DNA. Cells were harvested when ca. 90% confluent. Genomic DNA was extracted using the GenElute Mammalian Genomic DNA miniprep kit (Sigma-Aldrich). Integration sites were amplified by linker-mediated PCR as described previously [30]. Genomic DNA was digested using *MseI* and linkers were ligated (S1 Fig). Proviral-host junctions were amplified by nested PCR using Barcoded primers, generating 454 libraries. This enabled pooling of PCR products into one sequencing reaction. Products were gel-purified and sequenced using 454/Roche pyrosequencing (Titanium technology, Roche) on the 454 GS-FLX-instrument at the University of Pennsylvania. Reads were filtered based on perfect match to the LTR linker, Barcode and flanking LTR. All sites were mapped to the human genome requiring a perfect match within 3bp of the LTR end. Three random control sites were computationally generated and matched with respect to the distance to the nearest *Mse*I Cleavage site for each experimental site (matched random control, MRC). A more detailed explanation can be found in the supplementary guidelines of [63]. Normalization of experimental HIV-derived lentiviral vector sites to those of the MRC sites functions as a control for recovery bias due to cleavage by restriction enzymes. Analysis was performed as described previously and genomic heat maps generated using the INSIPID software (Bushman Lab, University of Pennsylvania). [30]. A detailed guide to interpret the heat maps presented can be found in [63]. The computation of DNase I site density was based on a table of DNase I sites obtained from [64]. Datasets used in the safe harbor analysis were retrieved from ENSEMBLE and/or UCSC (TxDB knownGenes, miRNA biotype, UCR; hg19) using BioMART [65]. The Allonco-list was used for oncogenes as published in [66]

## Results

### Generation of LEDGF-hybrids and stable cell lines

In an effort to distribute lentiviral vector integration more randomly over the genome, we modified LEDGF/p75, the cellular tether of the HIV Pre-Integration complex (PIC), by deleting the chromatin-reading PWWP-domain (ΔN_93_-LEDGF, Fig 1A and 1B) [32–34] relying on the remaining non-specific DNA-interacting regions in LEDGF_93-325_, such as the AT-hook domains and the CRs (Fig 1A; [39]). In addition, we generated LEDGF-hybrids where the PWWP-domain was exchanged with a set of alternative pan-chromatin recognition peptides of viral origin (Fig 1B, and Fig 2). Prototype Foamy Virus chromatin binding segment of Gag_534-546_ (PFV Gag_534-546_) [52–54], Human Papilloma Virus serotype 5 E2_242-257_, Human Papilloma Virus serotype 8 E2_240-255_ (HPV5 E2_242-257_ and HPV8 E2_240-255_, respectively)[56–58] and Kaposi’s Sarcoma Herpes Virus Latency Associated Nuclear Antigen_1-31_ (KSHV LANA_1-31_) [55] were used to replace the PWWP domain, generating ΔN_93_-LEDGF, PFV Gag_534-546_-ΔN_93_-LEDGF, HPV5 E2_242-257_-ΔN_93_-LEDGF, HPV8 E2_240-255_-ΔN_93_-LEDGF and KSHV LANA_1-31_-ΔN_93_-LEDGF fusions, respectively. All above-mentioned LEDGF-hybrids were used to complement LEDGF/p75-depleted cells (HeLaP4 (LEDGF_KD_) [60] and Nalm (LEDGF_KO_) cells [67]) employing SIV-based lentiviral vectors. As a positive control, cells were complemented with WT LEDGF/p75 (referred to as LEDGF/p75 back complementation (LEDGF_BC_)). In order to control for non-specific effects resulting from the expression of the fusion proteins we also generated stable cell lines expressing the respective chimeras carrying a D366N mutation in the LEDGF/p75 part, which abrogates the interaction with lentiviral integrase (IN) [68]. Protein integrity was corroborated by western blot analysis, with all LEDGF-hybrids migrating at the predicted molecular weights (S2 Fig). Of note, protein levels of PFV Gag_534-546_-ΔN_93_-LEDGF were lower in all experiments. Viability and growth rates of all cell lines were comparable to the parental HeLaP4 cells (data not shown).

### LEDGF-hybrids locate to the nucleus and display a distinct subnuclear distribution

**Fig 3:**
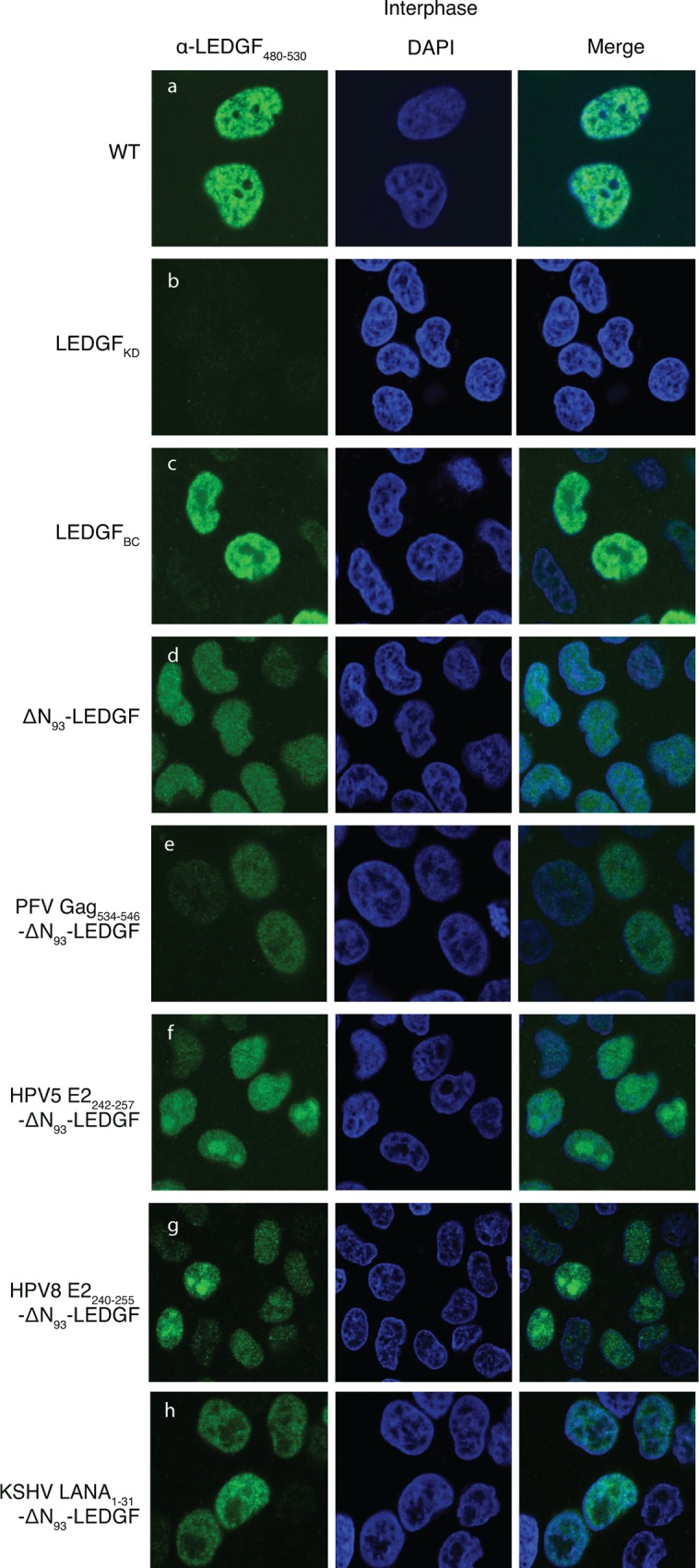
Subcellular localization of LEDGF/p75-hybrids in interphase cells. LEDGF/p75 depleted cell lines were complemented with the respective LEDGF-fusions. Laser scanning confocal images of HelaP4 cells, stained using an Ab recognizing LEDGF_480-530_, are shown in green. Nuclei were stained using Dapi (shown in blue). A merge of green and blue fluorescence is shown. Data depicted are representative for the respective cell lines. PFV, Prototype foamy virus; LANA, Latency associated nuclear antigen; HPV, Human papilloma virus; LEDGF, Lens epithelium-derived growth factor; DAPI, 4',6-Diamidino-2-Phenylindole.

In a first step, we evaluated the subcellular localization of the truncated ΔN_93_-LEDGF and the respective ΔN_93_-LEDGF-hybrids by immunocytochemistry (Fig 3). Complementation of LEDGF-depleted HeLaP4 cells (LEDGF_KD_) with LEDGF_BC_ resulted in a typical pattern of dense, fine speckles in the nucleoplasm excluded from the nucleoli during interphase (Fig 3C), phenocopying the endogenous LEDGF/p75 pattern (Fig 3A), which is in line with earlier reports [46]. Contrary, LEDGF/p75 lacking the chromatin-reading PWWP-domain exhibited a more diffuse nuclear distribution and located to the nucleoli as well (ΔN_93_-LEDGF, Fig 3D). In addition, all ΔN_93_-LEDGF peptide-fusions located to the nucleus (Fig 3E–3H), displaying a unique sub-nuclear distribution: the PFV Gag_534-546_- and the KSHV LANA_1-31_-fusion to ΔN_93_-LEDGF showed a punctate appearance in the nucleus and were excluded from nucleoli (Fig 3E and 3H), contrary to both HPV5 E2_242-257_- and HPV8 E2_240-255_-ΔN_93_-LEDGF fusions that were enriched in the nucleoli (Fig 3F and 3G). Similar subcellular distributions were observed for the respective cognate LEDGF_D366N_-hybrids (data not shown).

### LEDGF-peptide fusions rescue lentiviral vector transduction

**Fig 4:**
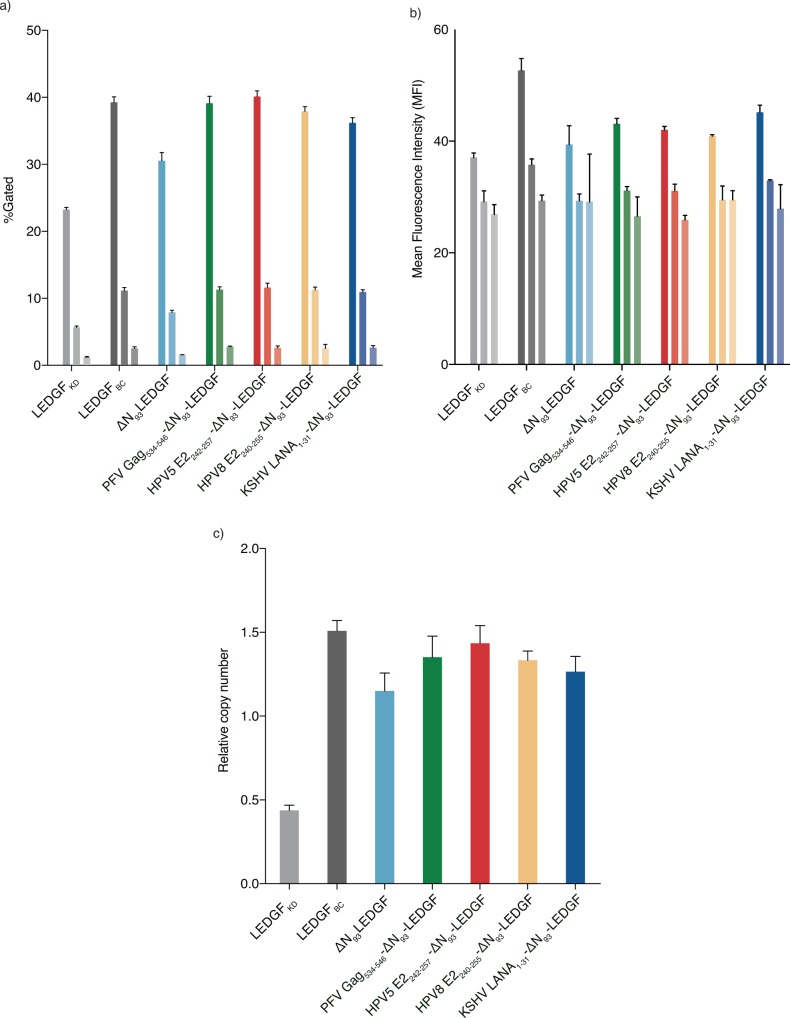
Rescue of lentiviral vector transduction by artificial LEDGF-hybrids. LEDGF-fusions were evaluated for their ability to support lentiviral vector transduction. LEDGF-depleted HelaP4-based cell lines stably complemented with LEDGF-hybrids were challenged with a VSV-G pseudo-typed lentiviral reporter vector encoding enhanced Green Fluorescent Protein (eGFP). Fluorescence was measured by fluorescence activated cell sorting and the different variables plotted:. (a) Percentage eGFP positive cells (transduction efficiency) and (b) Mean Fluorescence Intensity (MFI). Data are compiled for a representative experiment and depict averages of 3 replicates for 3 different vector dilutions (mean ± SD). (c) Lentiviral integrated proviral copies were determined by Q-PCR analysis on genomic DNA extracts of cells transduced with an MOI = 1. Data are represent the mean of 3 replicates ± SD. Statistical significance is calculated using a two-tailed t-test relative to LEDGF_KD_ or ΔN_93_-LEDGF. PFV, Prototype foamy virus; LANA, Latency associated nuclear antigen; HPV, Human papilloma virus; LEDGF, Lens epithelium-derived growth factor.

Next, we assessed whether the ΔN_93_-LEDGF-hybrids supported lentiviral vector transduction by complementing LEDGF-depleted cells (LEDGF_KD_) and employing wild-type LEDGF/p75 complemented cells (LEDGF_BC_) as control. The respective HeLaP4 cell lines were challenged with a dilution series of a lentiviral vector (multiplicity of infection (MOI) = 1, 0.2 or 0.04 (indicated in lighter colors)) encoding enhanced green fluorescent protein (eGFP) and firefly luciferase (fLuc) reporters [61]. Transduction efficiencies were determined by flow cytometry monitoring eGFP fluorescence (Fig 4A and 4B, showing transduction efficiency (eGFP positive cells; %Gated) and Mean Fluorescence Intensity (MFI), respectively). Complementation of LEDGF-depleted cells with LEDGF_BC_ restored transduction efficiency (Fig 4A) (***, p<0.005; two-tailed t-test relative to LEDGF_KD_), in line with earlier reports [46,69]. Complementation of LEDGF_KD_ cells with ΔN_93_-LEDGF, lacking the chromatin interacting PWWP-domain, partially rescued lentiviral transduction (78% compared to LEDGF_BC_) (Fig 4A, ***, p<0.005 compared to KD, two-tailed *t*-test). Addition of chromatin binding peptides to replace the PWWP domain displayed a significantly improved transduction relative toΔN_93_-LEDGF (***, p<0.005; two-tailed t-test), reaching efficiencies comparable to LEDGF_BC_ (Fig 4A). Similar results were obtained for different vector dilutions (Fig 4A) or when assessing complemented LEDGF/p75 knock-out cells (Nalm^-/-^, data not shown) [67] or when evaluating fLuc as a reporter (data not shown). Looking at Mean Fluorescence intensities all LEDGF-peptide fusions were about 20% lower than LEDGF_BC_ (Fig 4B). In addition to transduction efficiencies, we also determined the number of integrated copies (Fig 4C). Reintroduction of LEDGF/p75 (LEDGF_BC_) significantly improved vector integration (±-3.5-fold compared to LEDGF_KD_ (***, p<0.005, two-tailed *t*-test).

Likewise, ΔN_93_-LEDGF and all LEDGF-peptide fusions restored vector integration (2.5-fold more compared to LEDGF_KD_, ***, p<0.005, two-tailed *t*-test), albeit still to a lesser extent (reaching 68–74% of LEDGF_BC_, Fig 4B). The increased transduction efficiencies (%Gated) closely correlate with an increase in integrated viral vector copies (Fig 4C). Complementation with ΔN_93_-LEDGF alone, lacking any additional chromatin-tether, resulted in lower integrated copy numbers than ΔN_93_-LEDGF fused to chromatin engaging peptides (p-values<0.005, two-tailed t-test relative to ΔN_93_-LEDGF; 66.7% of LEDGF_BC_), supporting the notion that the chromatin-reading PWWP domain of LEDGF/p75 is not an absolute requirement for efficient integration.

### LEDGF-peptide fusions efficiently redistribute lentiviral integration

**Fig 5:**
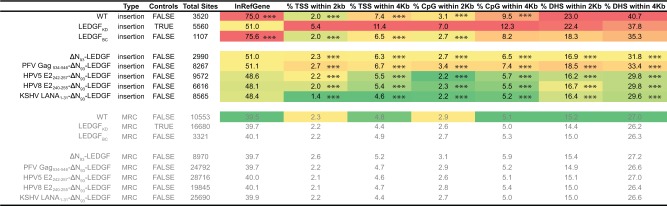
Integration frequency near genomic features. Fig showing the percentage HIV-derived lentiviral vector integration sites relative to features specific for integration viral vectors such as integration into the body of genes (Refseq genes, InRefGene), integration within 2kb-4kb windows near Transcription Start Sites (X5-end of genes, TSS), midpoint of CpG islands or DNase I-hypersensitive sites (DHS), counted in both the 5’ and 3’ direction. Dataset details are described in the MM section. Asterisks depict a significant deviation from LEDGF KD (two-tailed Chi-square test; ***, p-values <0.001). TSS, Transcription start sites; DHS, DNase I-hypersensitive sites; PFV, Prototype foamy virus; HPV, Human papilloma virus; KSHV, Kaposi’s sarcoma herpes virus; LANA, Latency associated nuclear antigen; MRC, matched random control.

**Fig 6:**
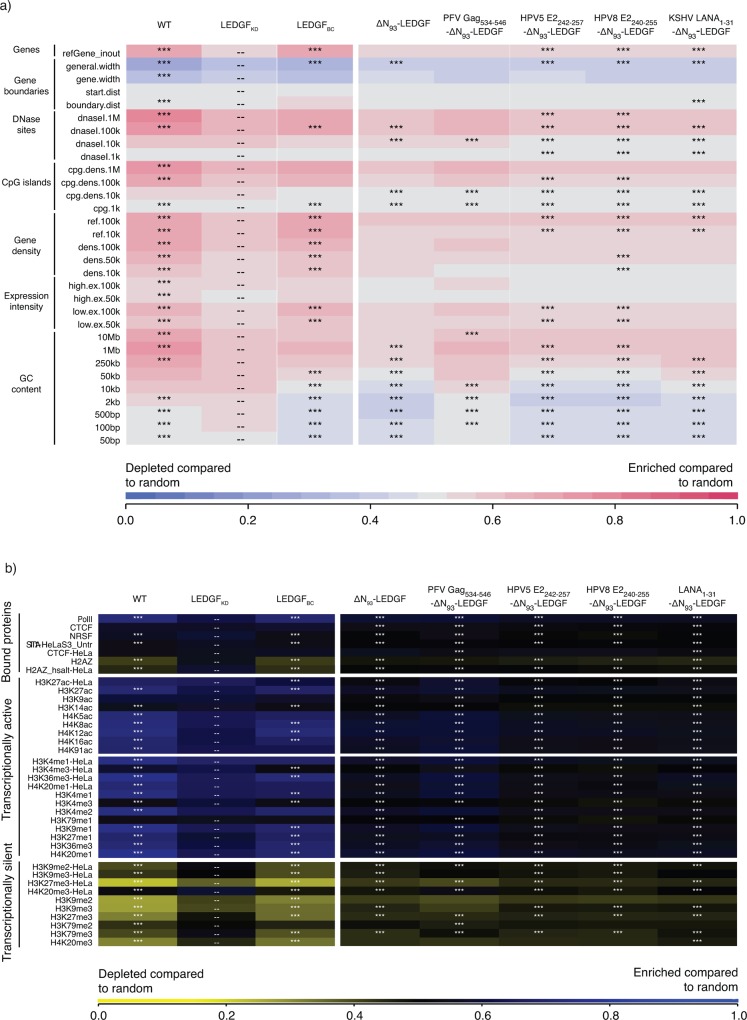
LEDGF-hybrids retarget lentiviral integration towards a more randomized pattern. (a) Genomic heat map comparing integration site data sets obtained from HeLaP4 LEDGF/p75 KD cells overexpressing different artificial LEDGF-hybrids to genomic features. Tile color depicts the correlation for an integration dataset with the respective genomic feature (left) relative to matched random controls, as indicated by the colored receiver operating characteristic (ROC) curve area scale at the bottom of the panel. Statistical significance (asterisks, ***p<0.001 ranked Wald tests) is shown relative to LEDGF KD population (double dash). Columns indicate different data sets, while rows indicate different genomic features analyzed (described in [63]). LANA, Latency associated nuclear antigen; HPV, Human papilloma virus; PFV, Prototype foamy virus; a; LEDGF, Lens epithelium-derived growth factor. (b) Epigenetic heat map comparing integration site data sets obtained from HeLaP4 LEDGF/p75 KD cells overexpressing different artificial LEDGF-hybrids to epigenetic features. Tile color depicting a positive or negative correlation to the respective epigenetic feature (10kb windows), relative to MRC, as indicated by the receiver operating characteristic (ROC) curve area scale at the bottom of the panel. Statistical significance (asterisks, ***p<0.001, ranked Wald tests) is shown relative to LEDGF KD population (dashed). Columns indicate different data sets while rows indicate different epigenetic features analyzed. Included features were limited to those identified in high-throughput studies HeLaP4 and primary CD4+ T-cells. Detailed information on epigenetic marks and their roles can be found in [87,88]. LANA, Latency associated nuclear antigen; HPV, Human papilloma virus; PFV, Prototype foamy virus; a; LEDGF, Lens epithelium-derived growth factor.

After showing that complementation of LEDGF/p75-depleted cells with ΔN_93_-LEDGF or any of the LEDGF-hybrids rescued vector integration, we determined the integration profiles in the respective cell lines. HIV-based viral vector integration sites were amplified and sequenced as described earlier [30,46], yielding a total of 62670 unique integration sites and their computationally generated matched random control (MRC) sites. Note that SIV-based viral vectors were used to complement LEDGF/p75-depleted cells, in order to avoid interference with the HIV-based viral vector integration site amplification and analysis. First, we analysed integration relative to a set of defined genomic features (Fig 5, Fig 6). Lentiviral vector integration in wild-type HeLaP4 cells (endogenous LEDGF/p75, Fig 6) is traditionally enriched in the body of transcription units (75.0% in RefSeq genes; Fig 5) but disfavoured transcription start sites (TSS) and promoter regions (2.0% within 2kb of the 5’ of a RefSeq gene and 3.1% within 2kb of a CpG island) [10,25]. LEDGF-depletion results in a more random integration site distribution, characterized by reduced integration into genes (51.0% in RefSeq genes) and increased integration close to TSS (5.4%) and CpG islands (7.0%), in line with previous work [30,46,70]. This phenotype was fully reverted upon LEDGF/p75 complementation (LEDGF_BC_; 75.6% in RefSeq genes). Comparable data were obtained for larger window sizes (only 2kb and 4kb are shown in Fig 5). Integration site distributions in cells expressing the respective LEDGF_D366N_ mutants were not different from LEDGF_KD_ cells (n = 16473; data not shown). Interestingly, the mere ablation of the PWWP domain (ΔN_93_-LEDGF) resulted in an overall more random distribution compared to LEDGF_KD_ cells, with decreased integration near retrovirus-specific features like gene bodies, TSS and promoter regions (*** p<0.001; χ2 test compared to LEDGF_KD_; Fig 5). Complementation of LEDGF-depleted cells with LEDGF-peptide fusions resulted in a comparable more randomized distribution (*** p<0.001; χ2 test compared to LEDGF_KD_; Fig 5). In a more elaborate analysis, we analysed global integration preferences and included a wide selection of genomic features, depicted as a genomic heatmap (Fig 6), comparing integration site data sets obtained from HeLaP4 LEDGF_KD_ cells to those of cells complemented with the respective LEDGF-hybrids. Tile color depicts the correlation for an integration dataset with the respective genomic feature (left) relative to matched random controls, as indicated by the colored receiver operating characteristic (ROC) curve area scale at the bottom of the panel. LEDGF/p75 depletion shifts integration out of transcriptionally active regions wich is reverted upon complementation with LEDGF/p75 (compare LEDGF_KD_ and LEDGF_BC_; shown in Fig 6), in line with previous data [30,46,70]. Cells complemented with ΔN_93_-LEDGF displayed an more randomly distributed integration profile, with tiles overall coloring less red or blue compared to LEDGF_KD_, integrating less near DNase sensitive regions, CpG-islands and GC-rich regions compared to LEDGF_KD_ (*** p<0.001, Wald statistics). Introduction of the heterologous HPV E2 and LANA_1-31_-peptide fragments to replace the PWWP-domain resulted in a ΔN_93_-LEDGF-like integration profile when compared to LEDGF_KD_ (p<0.001), whereas integration for PFV Gag_534-546_-ΔN_93_-LEDGF was less random. When displaying statistics relative to ΔN_93_-LEDGF (S3A Fig) integration frequencies near these genomic features is not significantly different between ΔN_93_-LEDGF and the respective ΔN_93_-LEDGF peptide-fusions, except for PFV Gag_534-546_-ΔN_93_-LEDGF (Fig 6). The reproducibility of the data observed for HPV5 E2_242-257_-ΔN_93_-LEDGF and HPV8 E2_240-255_-ΔN_93_-LEDGF complemented cell lines and the pronounced redistribution towards more random relative to LEDGF_BC_ (S3B Fig) underscores the effectiveness of LEDGF-based artificial tethers for retargeting of LV integration. Next to integration relative to genomic features, we also analyzed integration site densities near epigenetic features (Fig 6B). The epigenetic heat map displays yellow and blue tiles, with blue tiles indicating that integration frequency is enriched near these marks relative to MRC, whereas yellow tiles indicate that integration is disfavored compared to MRC. A near random distribution would result in a black tile. As reported previously, lentiviral integration correlates with histone marks associated with open and transcriptionally active chromatin (H3K4 mono-, di- and tri methylation, H3K14 and H4 acetylation, as well as acetylation and monomethylation of H3K9/K27/K79, H4K20 and H2BK5, …): [8] while disfavoring integration in transcriptionally silent regions or heterochromatin (H3K27me3, H3K9me3 or H4K20me3 and H3K79, respectively): [8] (WT; Fig 6). Depletion of LEDGF/p75 (LEDGF_KD_) resulted in a more random distribution (with tiles displaying a less pronounced blue or yellow color, and shifting towards black). This tendency was more outspoken for ΔN_93_-LEDGF and the HPV E2 and LANA_1-31_-peptide fusions compared to LEDGF_KD_ (Fig 6B), ΔN_93_-LEDGF (S4A Fig) or LEDGF_BC_ (S4B Fig), potentially because integration in LEDGF-depleted cells, at least in part, is tethered by HRP-2 [67].

### Artificial peptide-LEDGF/p75 hybrids result in a safer integration profile

**Fig 7:**
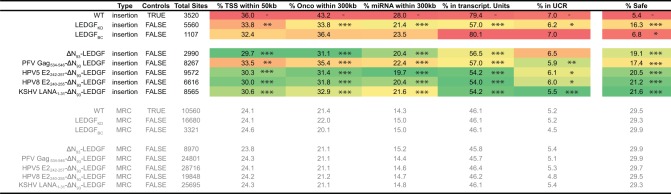
Integration frequency near safe harbor criteria. Fig showing the percentage HIV-derived lentiviral vector integration frequencies near features (TSS, Oncogenes [66], miRNA encoding regions, Transcription units and ultra conserved regions) that, when hit, are considered to be UNsafe as defined in [59] (Dataset details are described in the MM section). As such these features are used to define safe harbors as regions that fall outside these criteria. Percentages depict the fraction of integrations falling within the corresponding range relative to the criteria. The % of integrations negatively associated with these 5 features is used to calculate a safety profile. (*, p-value <0.5; **, p-value <0.05;***, p-value <0.005, Pearsons Chi-square compared to LEDGF_WT_ control). TSS, Transcription start sites; UCR, Ultra conserved regions; PFV, Prototype foamy virus; HPV, Human papilloma virus; KSHV, Kaposi’s sarcoma herpes virus; LANA, Latency associated nuclear antigen; MRC, matched random control.

Together, the presented above data indicate that lentiviral vector integration preferences are defined by LEDGF/p75 as a cellular tether, and are mostly dictated by the N-terminal PWWP-domain. The mere deletion of this domain, or replacement with alternative chromatin-interacting modules redistributes vector integration sites in a more random fashion. The question remains whether redistribution of proviral integration sites obtained for our LEDGF-hybrids also translated in a safer therapy, with a lower chance on insertional mutagenesis. In an effort to get a better view on the safety profile, we calculated integration frequencies near a specific set of previously defined criteria [59,66], such as transcription start sites (<50kb), oncogenes (<300kb) or miRNA coding regions (<300kb), transcription units and ultraconserved elements to define potentially unsafe integration events. The large window sizes impose a very stringent selection for lentiviral integration events away from these features, which in turn can thus be considered as more safe [59]. For each data set we evaluated the percentage of unsafe integrations (Fig 7 and S5 Fig) and in addition determined the percentage of safe sites (events not captured in any of the other criteria; Fig 7, % safe). When calculating the percentage in the parental cell line only 5.4% of all LV integration sites may be considered safe. LEDGF/p75-depletion results in a shift to 16.3% safe sites (p-value <0.005, Pearsons Chi-square compared to the LEDGF_WT_ control condition), a phenotype that was fully reverted upon LEDGF/p75_BC_ complementation (5.4%, no significant difference compare to LEDGF_WT_). Ablation of the N-terminal PWWP-domain again boosted the percentage safe integration events to 19.7% (p-value <0.005, Pearsons Chi-square compared to the LEDGF_WT_ control condition). Addition of heterologous peptide fragments KSHV LANA_1-31_ and HPV8 E2_240-255_ to the N-terminal end of LEDGF_93-530_ slightly increased the % safe integrations relative to ΔN_93_-LEDGF (S5A Fig) with complementation up to 20.2% for HPV5 E2_242-257_-ΔN_93_-LEDGF, 21.2% for HPV8 E2_240-255_-ΔN_93_-LEDGF and 21.6% for KSHV LANA_1-31_-ΔN_93_-LEDGF when considering these criteria (p-value <0.05, Pearsons Chi-square compared to the ΔN_93_-LEDGF control condition). Relative to the LEDGF_BC_ condition our LEDGF-chimera increased the percentage of safe sites more than 3 fold (p-value <0.005, Pearsons Chi-square, S5B Fig). Of note, for the MRC conditions, we obtained a maximum of 30% integrations in safe harbors.

## Discussion

Integration of retroviral vectors into the host cell genome makes them invaluable tools for gene therapeutic applications where life-long correction is key. Previous reports showed effective gene transfer enabling long-term gene correction (For a review see [71]). However, severe adverse events in these clinical studies (using full-LTR driven gamma-retrovirus vectors) raised serious concerns regarding the safety of gene therapy when using integrating vectors (derived from the family of retroviruses) [14,15]. The yRV preference for integration into enhancer regions and concomitant activation of proto-oncogenes led to malignant transformation of cells and clonal expansion [10–12]. Therefore, multiple studies have been triggered to increase the safety of the used retroviral vectors, which include the use of other subtypes (lenti or alpha instead of gammaretroviral), SIN-LTR design [72–75], tissue specific promoters [76], changing integration properties [45–47] and insulator sequences as enhancer and silencer blockers [77]. Meanwhile, lentivirus vectors became the mean of choice when using retroviruses for gene transfer and clinical gene therapy due to their safer integration profile and lower genotoxicity in preclinical models. As such, any successful modification avoiding an increased integration of these vectors into gene coding regions may be relevant for translation into the clinics. Stable integration however will always imply the intrinsic risk of vector-induced genomic perturbation, open reading frame-disruption, leading to loss of function or transcriptional deregulation of neighbouring genes as indicated by the report on SIN-LV affected splicing [78]. In addition, also LV integration may lead to clonal dominance as reported in the beta-thalassemia trial, which could be an indicator of upcoming malignant transformation [19]. Therefore it is important to gain additional mechanistic insights into the molecular mechanism of integration and integration site selection for LVs to be accepted for general therapeutic use. We and others substantially contributed to the elucidation of the role of LEDGF/p75 as a molecular tether of lentiviral vector integration. As a cellular cofactor of lentiviral integration, LEDGF/p75 orchestrates lentiviral integration preference by binding H3K36me3 in the body of active transcription units via its N-terminal PWWP domain, but it is the vector-encoded integrase that catalyzes the integration reaction. Depletion of LEDGF/p75 by knockdown or knockout strategies shifts lentiviral vector integration out of active genes, yet integration is not completely random [67,79], which at least in part can be explained by residual targeting via HRP-2 [67]. Here we set out to study whether different LEDGF-hybrids could be generated to distribute lentiviral integration sites more randomly. This line of vector development is based on the further increasing interest in new vector platforms displaying a close-to-random insertional profile potentially reducing the probability of proto-oncogene activation lowering the genotoxic potential [51,80,81]. In an effort to achieve a more random integration site distribution, we deleted the specific chromatin-binding PWWP module of LEDGF/p75 (aa 1–93), or we replaced it with alternative pan-chromatin binding modules. In case of LEDGF/p75, it is demonstrated that the PWWP domain recognizes H3K36me3, a chromatin mark that is particularly enriched in the body of active transcription units [32–36]. Complementation of LEDGF-depleted cells with a LEDGF/p75-protein that had its PWWP domain deleted (ΔN_93_-LEDGF) or replaced with alternative chromatin binding modules showed unique subnuclear distributions for each of the constructs, indicating that these deletion of the PWWP domain, or the replacements with any of the other peptides, resulted in a specific redistribution within the nuclear compartment of the artificial LEDGF chimera (Fig 3). The latter phenotype can be attributed to the AT-hook motifs and charged regions present in the N-terminal end of ΔN_93_-LEDGF, together with the specific peptides that replaced the PWWP domain. After working up integration sites, analysis showed that lentiviral integration preferences for most of the constructs resulted in a more random distribution than under LEDGF depleted conditions (genomic and the epigenetic heat map representations; Fig 6A and 6B), except for PFV Gag_534-546_-ΔN_93_-LEDGF. For example, in the latter cells LV integration was still enriched near epigenetic markers for transcriptionally active chromatin, albeit less outspoken than observed with LEDGF_WT_ and LEDGF_BC_ cells (S4B Fig). Interestingly, peptide addition was not required to obtain a more random distribution. Lentiviral integrations in ΔN_93_-LEDGF expressing cells were redistributed in a fairly random manner, with tile colors shifting to grey and black (for the genomic and the epigenetic heat map representations, respectively) indicating that integration frequencies for these features are not enriched nor depleted compared to the matched random integration site distribution. Comparison with LEDGF KD shows that integration is more randomly distributed than under LEDGF depletion (*** p<0.001, Wald statistics; Fig 6A and 6B). Fusion of short pan-chromatin binding peptides to the truncated ΔN_93_-LEDGF resulted in similar shifts towards a more randomized integration profile. The fact that all peptide fusions display a unique subnuclear location, suggest that their interaction with chromatin is different. Even though the overall integration frequencies are highly similar (considering the genomic and the epigenetic features analyzed), larger integration site datasets (>10e5 sites) would be required to allow more detailed analysis on the specific subsets. In an effort to estimate the effect of the more randomized distribution on safety, we calculated the frequency of integration relative to a set of safe harbor criteria for the individual integration site datasets [59]. This analysis showed that the more random distributions resulted in a lower genotoxic profile with 18–22% of integrations meeting safe harbor criteria for our LEDGF-chimera compared to only 5.4% for cells carrying wild-type LEDGF/p75, all LEDGF-chimera resulted in a safer distributions over the genome. Fully targeted integration towards safe harbor regions like the *AAVS1* or *CCR5* locus would be the ultimate solution to circumvent insertional mutagenesis [59,66,82]. Several methods for site-directed gene correction have been developed using genetic scissors based on Zinc-finger nucleases, transcription activator like effector nucleases or more recently RNA-guided nucleases (CRISPR/Cas9) (for a review [83]). However, site directed integration would no doubt impair transduction efficiencies. Our approach improves the therapeutic potential of lentiviral vectors by decreasing the risk/benefit ratio, still supporting high transduction efficiencies. The fact that integration can be directed to genomic regions that are not targeted under wild-type conditions nor LEDGF-depleted conditions, indicates that integration in these areas is disfavored due to the absence of a tether, rather than the presence of specific obstacles such as steric hindrance resulting from the condensed chromatin structure. As an alternative to the generation of stable cell lines as employed here, we demonstrated earlier that mRNA-electroporation ensures timely, high-level recombinant protein expression that is sufficient to retarget lentiviral vector integration [48]. When combined with IN mutant lentiviral vectors that selectively bind complementary LEDGF/p75 variants [84], this approach should be broadly applicable to introduce therapeutic or suicide genes for cell therapy, such as genetic modification of patient-specific iPS cells and improve safety of lentiviral vectors. With the occurrence of potential adverse effects being of multi-factorial nature [85] novel therapeutic approaches should be evaluated in relevant functional assays able to predictively assess the cytotoxicity observed *in vivo* [86], a continuous effort aiming at abolishing the risk of insertional mutagenesis will be required for gene therapy to become a broadly accepted treatment alternative.

## Supporting Information

S1 Fig Oligo sequences used in this study. Fig depicting the different oligos used in this study together with their nucleotide sequence.
(EPS)

S2 Fig Western analysis of LEDGF-fusions. LEDGF depleted cell lines were complemented with the respective LEDGF-hybrids. Total cell lysates were prepared and separated on a 12,5% SDS gel. An antibody recognizing LEDGF_325-530_ was used for detection. β-tubuline detection was used as an equal loading control. WT, Wild type; KD, Knockdown; PFV, Prototype foamy virus; LANA, Latency associated nuclear antigen; HPV, Human papilloma virus; LEDGF, Lens epithelium-derived growth factor.
(EPS)

S3 Fig LEDGF-hybrids retarget lentiviral integration towards a more randomized pattern. Genomic heat maps comparing integration site data sets obtained from HeLaP4 LEDGF/p75 KD cells overexpressing different artificial LEDGF-hybrids to genomic features. Tile color depicting the nature of the correlation for an integration dataset with the respective genomic feature (left) relative to matched random controls, as indicated by the colored receiver operating characteristic (ROC) curve area scale at the bottom of the panel. Statistical significance (asterisks, ***p<0.001, ranked Wald tests) is shown relative to (a) ΔN_93_-LEDGF or (b) LEDGF_BC_, respectively (double dash). Columns show different data sets while rows indicate different genomic features analyzed (described in [63]). LANA, Latency associated nuclear antigen; HPV, Human papilloma virus; PFV, Prototype foamy virus; LEDGF, Lens epithelium-derived growth factor.
(EPS)

S4 Fig LEDGF-hybrids retarget lentiviral integration towards a more randomized pattern. Epigenetic heat map comparing integration site data sets obtained from HeLaP4 LEDGF/p75 depleted cells overexpressing different artificial LEDGF-hybrids to epigenetic features, generated using the INSIPID software (Bushman Lab, University of Pennsylvania). Tile color depicting a positive or negative correlation to the respective epigenetic feature (10 kb windows), relative to matched random controls, as indicated by the receiver operating characteristic (ROC) curve area scale at the bottom of the panel. Statistical significance (asterisks, ***p<0.001; ranked Wald tests) is shown relative to (a) ΔN_93_-LEDGF or (b) LEDGF_BC_, respectively (double dash). Significance is reached when p<0.001, compared to MRC. Columns indicate different data sets while rows indicate different epigenetic features analyzed. Included features were limited to those identified in high-throughput studies performed in HeLa and primary CD4+ T-cells. Detailed information on epigenetic marks and their roles can be found in [87,88]. LANA, Latency associated nuclear antigen; HPV, Human papilloma virus; PFV, Prototype foamy virus; a; LEDGF, Lens epithelium-derived growth factor; MRC, matched random control.
(EPS)

S5 Fig Integration frequency near safe harbor criteria. Fig showing the percentage HIV-derived lentiviral vector integration frequencies near features (TSS, Oncogenes [66], miRNA encoding regions, Transcription units and ultra conserved regions) that, when hit, are considered to be unsafe as defined in [59] (Dataset details are described in the MM section). As such these features are used to define safe harbors as regions that fall outside these criteria. Percentages depict the fraction of integrations falling within the corresponding range relative to the criteria. The % integrations negatively associated with these 5 features is used to calculate a safety profile. (*, p-value <0.5; **, p-value <0.05;***, p-value <0.005, Pearsons Chi-square compared to (a) ΔN_93_-LEDGF or (b) LEDGF_BC_ control condition). TSS, Transcription start sites; UCR, Ultra conserved regions; PFV, Prototype foamy virus; HPV, Human papilloma virus; KSHV, Kaposi’s sarcoma herpes virus; LANA, Latency associated nuclear antigen; MRC, matched random control.
(EPS)
